# Nuggets

**Published:** 1890-12

**Authors:** C. Carleton Smith

**Affiliations:** Philadelphia


### "NUGGETS."

C. Carleton Smith, M. D., Philadelphia.

In treating children who are too young to make known the nature of their ailments, the objective symptoms are more important than the subjective symptoms. We have to watch keenly in order to select the remedial agent.

There are eight remedies that have the characteristic symptom " wants to be carried in arms." These are Cham., Ant-tart., Arsen-alb., Cina, Ignatia, Kali-c., Puls., and Brom. The Cham. child cries almost constantly. And this crying ceases when the child is carried quietly up and down the room, the cries returning the moment the motion ceases, which latter makes the child so angry that it will viciously slap the nurse.

The Ant-t. child wants to be carried only by its mother, but no one else. And if another person approaches or touches it it will scream.

The Cina child's irritability simulates very closely that of Cham., with this difference: If the mother becomes weary of carrying it it can be comforted by fast rocking.

The Ars. child wants to be carried fast. Also the Brom. child.

The Puls. child wants to be carried slowly.

The Ignatia child is not cross, but rather too affectionate. Yawns all the time spasmodically. And wakes out of a very light sleep screaming and trembling all over.

The Kali-carb. child is peevish and irritable, but not to a great degree. And when he is touched he does not scream as under Ant-t., but he starts suddenly as if in alarm.

Aconitum may be added to this list under amelioration from fast rocking, especially in abnormal conditions following sudden fright.

When a child habitually, after nursing the breast, cries for water, and invariably throws it up, give the little one Arnica.

In hydrocephalus, when the child's arms below the elbows become deathly cold, think of Arnica.

When a patient in his illness hears everything but the human voice, study Arsenicum.

If he hears nothing but the human voice, do not forget Ignatia.

With regard to Arsenicum we must not forget Dr. Lippe's observation, that the pulse of this drug is more rapid in the morning than in the evening. And this characteristic also corresponds with Sulphur.

Arsenicum has also aggravation from sea bathing. Also aggravation from rest in bed, but amelioration from warmth of bed. Reverse this exactly, and we have the Mercurius condition.

A characteristic symptom of Arum-triphyllum which I observed many years ago is this: The patient complains that the nose is completely stopped up. And yet there is a constant fluid discharge from it which has to be wiped away continually. This condition I have observed in cases of scarlet fever, and Arum. was the curative drug.

When a patient complains that every time he urinates his piles come down, give him Baryta-carb.

Another peculiar symptom belonging to Bar-carb. is a numb feeling creeps up from the knees to scrotum and penis, disappearing as soon as he sits down.

Under Bismuth we have a symptom exactly similar to Ars., viz., water is vomited as soon as it reaches the stomach. Bismuth also has marked restlessness, like Ars., but the Bismuth patient vomits *all* fluids, which is not the case under Ars. And again, under Bismuth the patient will eat his rations for several days in succession, and then begin to vomit, keeping it up for a whole day.

In pneumonia the Bryonia sputa is of a soft brick shade in color, is quite tough, and falls like lumps of jelly.

The Bryonia pains are made better by lying on the painful side, and worse by lying square on the back. In pregnancy she cannot drink water, for the sight of it causes her to vomit. If she bathes she must close her eyes while in the water. The Phos. patient when coughing holds his abdomen with both hands. The acute chest-pains of Phos. are generally worse on right side, and aggravated by the least pressure in the intercostal spaces, and lying on right side.

Phosphorus has also, as a skin symptom, little ulcers outside of large ones—some healing and others healed. Cuprum-aceticum has a symptom exactly like Lachesis, and will, therefore, have to be compared with this latter remedy in practice. It is this, protrusion and retraction of the tongue like a snake. In epilepsy, where Cuprum-acet. is curative, the aura begins at the knees, ascending until it reaches the hypogastric region, when unconsciousness occurs, with foam at the mouth, and falling down convulsed. Another very important red thread which we find in the study of this drug, is that as soon as the patient goes into a high-ceiling room the head reels and she loses consciousness.

A characteristic of Cyclamen is as follows: After delivery patient complains of severe colicky bearing-down pains, each pain accompanied with a gush of blood, which latter causes momentary relief only.

When children cough at night while asleep without awaking, Cyclamen may be the remedy.

In severe diseases patients sometimes wake up during the night with hands feeling twice their natural size, so that they cannot make any use of them (is a key-note for Aranea-diadema). When drinking very cold water or eating ice-cream, patients complain of sharp pain in forehead, extending down into the nose. Give such cases Digitalis. Also under this remedy the patient feels as if heart would stop beating if he dared to move. Gelsemium has just the opposite. Patient must move in order to prevent the heart from ceasing to beat.

A symptom of Euphrasia worth recording is that when the patient is exposed to a south wind she is sure to have her cough aggravated.

The Euphrasia patient is better in open air. Same as Pulsatilla. Worse when in-doors.

Sensation as if vision was obscured by smoke. Study both Gelsemium and Crocus.

In intermittent fever the patient under Gels. wants to be held during the shake. Under Lachesis he wants some one to lie right across his body. In severe illness, when a patient's tongue, upper and under surface, is completely coated a decided *bluish-white*, the quicker you give him Glonoine the better. Look to Guaiacum in violent spasmodic inflammatory action of the larynx with such violent palpitation of the heart as to almost cause suffocation.

